# Similar Individual Serum Levels of MCP‐1, TNF‐α, and IL‐6 From Periodontitis Patients Before and 1 Year After Treatment

**DOI:** 10.1111/sji.70094

**Published:** 2026-01-24

**Authors:** Teun J. de Vries, Sergio Bizzarro, Irene Di Ceglie, Doran Y. Sol, Peter L. E. M. van Lent, Ton Schoenmaker

**Affiliations:** ^1^ Department of Periodontology, Academic Centre for Dentistry (ACTA) University of Amsterdam and Vrije Universiteit Amsterdam the Netherlands; ^2^ Department of Rheumatology Radboud University Nijmegen the Netherlands; ^3^ IRCCS Human Research Hospital Milan Italy

**Keywords:** cytokine equilibrium, cytokine signature, IL‐6, MCP‐1, osteoclast, periodontal ligament fibroblasts, periodontitis, serum, TNF‐α

## Abstract

Treatment of periodontitis may lower the inflammation status as measured in sera from patients. To investigate this in a biological assay, we co‐cultured periodontal ligament fibroblasts (PDLFs) from a healthy donor and peripheral blood mononuclear cells (PBMCs) as a source for osteoclasts. PDLFs were primed for 3 days with serum from periodontitis patients before and from 1 year after successful treatment of periodontitis (*n* = 13). After priming, PBMCs were added. No significant differences in the number of osteoclasts that formed after 3 weeks were found between baseline and after‐treatment serum priming. To find an explanation, we analysed the serum composition on the presence of inflammatory, pro‐osteoclastogenic cytokines. We found no differences between before and after treatment for cytokines MCP‐1, TNF‐α, and IL‐6. Interestingly, these levels of cytokines at baseline and after treatment strongly correlated per individual (correlation *p*‐value between *p = 0.03* and *p < 0.0001*, correlation *r* ^2^ between 0.37 and 0.89). Strikingly, between each other, MCP‐1, TNF‐α, and IL‐6 did not correlate. These results show that over the 1‐year time frame a cytokine equilibrium, the relatively similar levels of cytokines over a longer period, exists that is unique per individual. This suggests that the immune system is a constant for these cytokines, adapted to the needs of the individual. Larger cohorts, the inclusion of healthy individuals and longer time intervals need to be studied to further establish the true magnitude of these intriguing findings.

## Graphical abstract

Periodontitis patients before and 1 year present with similar levels of MCP‐1, TNF‐α, and IL‐6, cytokine equilibrium. MCP‐1, TNF‐α, and IL‐6 are not correlated per individual, meaning that some individuals may present with relatively high levels of one of the cytokines with lower levels of the other cytokines or cytokine signature.

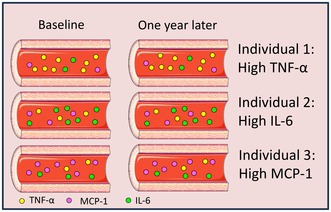

## 1 Introduction

Periodontitis is an inflammatory disease ultimately leading to alveolar bone loss and loss of teeth. During the progression of the disease, local stimulation by products of periopathogenic bacteria evokes a local immunological response where cytokines such as interleukin‐1 (IL‐1), IL‐6, IL‐17, monocyte chemoattractant protein‐1 (MCP‐1, also known as CCL‐2), and tumour necrosis factor‐α (TNF‐α) are being produced [1, 2, 3]. These molecules may locally induce production or increase the activity of the bone‐degrading cells, the osteoclasts, and thus exacerbate the pathological alveolar bone loss. Successful treatment of periodontitis results in a reduction of periodontal inflammation and prevention from further loss of alveolar bone, suggesting lower osteoclast number or activity.

Presence and activity of osteoclasts define periodontitis and set it apart from gingivitis. Osteoclasts are multinucleated cells that originate from the fusion of myeloid precursor cells present in blood and bone marrow. The attraction toward the inflamed area and subsequent fusion of these precursor cells into multinuclear osteoclasts is mediated by local cells, for instance by periodontal ligament fibroblasts (PDLF) that align alveolar bone and the tooth root [4]. The inflamed periodontium produces a gradient of chemo‐attractants and cytokines such as IL‐6, MCP‐1 and TNF‐α that all have a role in the attraction of leukocytes into the inflamed tissue and in the local priming of cells [1]. During periodontitis, the levels of cytokines to which these PDLF in situ are exposed are increased, which results in an increased induction of osteoclastogenesis.

It is highly conceivable that systemically, serum levels of these cytokines during an active inflammatory disease are higher than following successful treatment. As such, higher levels in serum may prime leukocytes before they leave the circulation [5]. Rheumatoid arthritis (RA) is such a disease, where patients with active disease have higher levels of inflammatory cytokines in the circulation compared to patients that are in remission [6]. Osteoclast precursors in peripheral blood from both rheumatoid arthritis and periodontitis display an increased capacity to differentiate into osteoclasts [6]. Remarkably, rheumatoid arthritis patients that were treated with anti‐TNF showed an improved periodontal status after months of therapy, indicating common pathways between the two diseases [7]. Osteoblasts stimulated with serum of active RA gave rise to more osteoclasts compared with osteoblasts that were stimulated with serum from patients in remission. Though likely a cocktail of cytokines caused this effect, specific inhibition during priming of the osteoblasts with anti‐IL‐6 and osteoprotegerin (OPG) diminished the number of osteoclasts in subsequent cocultures with PBMCs, specifically in active RA [8].

Many patients may accumulate various medical conditions simultaneously, influencing the serum levels of cytokines [9, 10]. Since periodontitis is associated with many comorbidities [11], one must select periodontitis patients without self‐reported systemic comorbidities when investigating sera before and after treatment. In this study, we aimed to investigate the priming effect of PDLFs of periodontitis patients' sera on osteoclast formation. To the best of our knowledge, this has never been studied. It is clinically relevant, since an inflammatory phase is followed by a phase of tissue recovery when periodontitis is successfully treated. To study the effect of the alleged lower inflammatory composition of the sera of successfully treated periodontitis patients, we selected periodontitis patients who responded best to treatment. This patient group could represent then the largest effect in sera before and after treatment. Sera before and 1 year after successful treatment were selected. Periodontal ligament fibroblasts, that stimulate osteoclast formation in vitro [4], were primed with these paired sera (at baseline and 1 year after successful treatment). Subsequently, osteoclast formation was assessed by seeding peripheral blood mononuclear cells as a source for osteoclast precursors onto the primed fibroblasts. As reviewed by Sokos et al. [4], PDLFs interact with PBMCs to provide the osteoclast differentiation signals to PBMCs, since no or very few osteoclast‐like cells are formed in the absence of PDLFs. We hypothesize that periodontal ligament fibroblasts that were exposed to serum at baseline would give rise to higher numbers of osteoclasts when cocultured with osteoclast precursors. As a secondary aim and in order to explain osteoclastogenesis findings, we analysed the serum levels of inflammatory and anti‐inflammatory cytokines that are associated with osteoclast formation.

## 2 Materials and Methods

### 2.1 Sera Periodontitis Patients

The sera from the patients were obtained from patients previously recruited [12]. Unique for this study protocol was that patients only had chronic periodontitis and did not present with other comorbidities: diabetes, diagnosis of metabolic syndrome, cardiovascular disease, (auto)immune disease or any other systemic or metabolic disease, and not receiving any medication for hypertension, dyslipidemia or hyperglycemia [12]. All patients received non‐surgical periodontal treatment, carried out by three experienced and specifically trained dental hygienists of the Department of Periodontology at ACTA in three appointments within 1 week. After completion of the active therapy, all patients were subsequently enrolled in a 3‐monthly maintenance program at the Department of Periodontology until the end of the follow‐up (1 year). When designing the osteoclastogenesis experiment, sera from the 13 patients that responded best to treatment were selected deliberately as a starting point, since these theoretically would have most deviant serum parameters. Attachment loss was used as a parameter to assess clinical improvement and to rank patients from bad to best responders. Other periodontal parameters that were assessed were: pocket depth, clinical attachment loss, bleeding on probing and plaque percentage. Sera were taken at *t* = 0 (intake) and after 1 year of treatment.

### 2.2 Periodontal Ligament Fibroblast Culture

PDLFs were obtained from an extracted third molar from a patient in their early twenties. The surrounding tissues did not show any signs of inflammation. Written informed consent was obtained as required for surgical waste material. By means of a scalpel, fragments of periodontal ligament were obtained from the middle third of the roots. PDLFs grew out of these scraped tissue fragments and were expanded for three passages and stored in liquid nitrogen [13].

### 2.3 Human Peripheral Blood Mononuclear Cells Isolation

PBMCs were isolated from one buffy coat, as the source of osteoclast precursor cells (Sanquin, Amsterdam, The Netherlands) as previously described [13]. The buffy coat was diluted 1:1 in PBS containing 1% citrate buffer. Twenty‐five millilitres of diluted blood was then layered on 15 mL lymphoprep (Axisshield Po CAS, Oslo, Norway) and centrifuged for 30 min at 1200 g without brake to separate PBMCs from red blood cells and granulocytes. The interphase containing PBMCs was collected and resuspended in PBS 1% citrate buffer. PBMCs were washed at least five times in PBS 1% citrate buffer and finally recovered in DMEM (Gibco BRL, Paisley, Scotland) containing 10% Fetal Clone 1, a type of fetal calf serum (HyClone, Logan, UT) and 1% antibiotics (Antibiotic antimycotic solution, Sigma, St. Louis, MO).

### 2.4 Co‐Cultures

PDLFs from one patient were defrosted from liquid nitrogen and used in the experiments at passage 4. 1.5*10^4^ PDLF's per well were seeded in 48 well plates with 400 μL DMEM +10% Fetal Clone 1 (Hyclone, Logan, UT) + 1% PSF. After 3 days of priming with 10% human serum from the 13 donors, for each donor baseline and 1 year after treatment serum was used. The medium was removed and 5*10^5^ viable PBMCs (assessed using Muse Cell Analyser using Muse Count and Viability kit, Merck Millipore, MA, USA) in DMEM +10% FC1 and 1% PSF per well were added on top of the PDLFs. The culture medium was refreshed every 3–4 days. The culture was analysed for osteoclast formation after 21 days of co‐culture. In the used experimental design of a co‐culture, the variables were the 13 paired donor sera. The PDLFs from one donor and the PBMCs could be regarded as 2 tester cell lines.

### 2.5 TRACP‐Staining and Osteoclast Quantification

After 21 days, the cultures were washed with PBS and fixed in paraformaldehyde. TRAcP staining was performed using the TRAcP kit (Sigma, # 387A‐1kt) according to the manufacturer's protocol. The nuclei were stained with diamidino‐2‐phenylindole dihydrochloride (DAPI).

The osteoclast quantification was performed by counting all multinucleated TRAcP‐positive cells with three or more nuclei under the fluorescence microscope (Leica, Wetzlar, Germany); the average of duplicate wells was used. The entire well was assessed.

### 2.6 RNA Analysis and PCR

RNA isolation was performed on samples directly after the 3 day priming and after 14 days with PBMCs using the QIAGEN RNeasy kit (Qiagen, Hilden, Germany). cDNA synthesis was performed using the Fermantis cDNA synthesis kit (Thermofischer Scientific, Waltham, MS, USA). qPCR was performed on the ABI7000 sequence detection system (Applied Biosystems, Foster city, CA, USA) using SYBR Green. After an initial activation step for 10 min at 95°C, 40 cycles of a standard 2‐step qPCR protocol (95°C for 15 s, 60°C for 1 min) was on the SDS7000 QPCR machine (Applied biosystems, Foster City, CA). 5 ng cDNA was used in a total volume of 20 μL containing SYBR green master mix (applied biosystems) and 1 μM of each primer. All primers are exon spanning (Table 1).

**TABLE 1:** Primer sequences used for PCR.

Gene	Sequence 5′‐3	Amplicon length (bp)	Ensemble gene ID
*β2 microglobulin*	AAGATTCAGGTTTACTCACGTC	293	ENSG00000166710
	TGATGCTGCTTACATGTCTCG		
*TRAcP*	CACAATCTGCAGTACCTGCAAGAT	128	ENSG00000102575
	CCCATAGTGGAAGCGCAGATA		
*DCSTAMP*	ATTTTCTCAgTgAgCAAgCAgTTTC	101	ENSG0000016493
	AGAATCATGGATAATATCTTGAGTTCCTT		
*IL‐1β*	CTTTGAAGCTGATGGCCCTAAA	100	ENSG00000125538
	AGTGGTGGTCGGAGATTCGT		
*IL‐6*	GGCACTGGCAGAAAACAACC	85	ENSG00000136244
	GGCAAGTCTCCTCATTGAATCC		
*IL‐17*	TCCTGGGAAGACCTCATTGG	100	ENSG00000112115
	AATTTGGGCATCCTGGATTTC		
*M‐CSF*	CCGAGGAGGTGTCGGAGTAC	100	ENSG00000184371
	AATTTGGCACGAGGTCTCCAT		
*RANK*	CCTGGACCAACTGTACCTTCCT	67	ENSG00000141655
	GCCCAACCCCGATCATG		
*RANKL*	CATCCCATCTGGTTCCCATAA	60	ENSG00000120659
	GCCCAACCCCGATCATG		
*OPG*	CTGCGCGCTCGTGTTTC	100	ENSG00000164761
	ACAGCTGATGAGAGGTTTCTTCGT		
*TNF‐α*	CCCAGGGACCTCTCTCTAATCA	103	ENSg00000111956
	GCTTGAGGGTTTGCTACAACATG		

Expression of housekeeping gene beta2 microglobulin (B2M) was not affected by the experimental conditions. Samples were normalised for the expression of B2M by calculating the ΔCt (Ct, gene of interest—Ct, B2M). Subsequently, the relative expression of the different genes was expressed as 2^−(ΔCt)^. Data analysis was performed on these relative expression data.

### 2.7 Luminex

For further interpretation of results of the osteoclast formation results, we analysed various cytokines that play a role in the formation of osteoclasts. We determined levels of both anti‐osteoclastogenesis (IFNγ, IL‐4, IL‐10) and pro‐osteoclastogenesis cytokines (IL‐1β, IL‐6, IL‐8, IL‐17, MCP‐1 and TNFα) [14, 15]. Sera at baseline and 1 year after periodontal treatment were measured on the Bio‐Plex 100 by Bio‐Rad with Luminex multi‐analyte technology, using Milliplex cytokine kits (Millipore, Billerica, MA). The Luminex assays were performed according to the manufacturer's instructions. Concentrations were read in a standard curve per cytokine. All read‐outs lower than the detection limit were scored “0”. The samples were coded; the operator was not aware of the donor or the time point of serum sampling.

### 2.8 Statistical Analysis

Paired *t*‐tests were used to compare the means between 2 matched groups. We used this to compare the number of osteoclasts before and after treatment and when analysing the gene expression differences in the before and after groups. Linear regression analysis was performed to analyse correlations of cytokine levels of the same patient before and after treatment. *p*‐values < 0.05 were considered significant. All data underwent a normality test. The *n* = 13 gave rise to normally distributed data sets for all parameters. Statistical analysis and the making of the graphs was performed with the GraphPad Prism, version 8. For the PCR analysis, the relative expression was used, which is calculated as 2^−ΔCt^. Since the data for PCR were not normally distributed, Wicoxon signed rank test was used.

### 2.9 Ethics Statement

This randomised clinical trial and subsequent analysis of its materials was approved by the medical ethical committee from the Academic Medical Centre of Amsterdam, The Netherlands (MEC 07/264).

## 3 Results

### 3.1 Periodontal Treatment Results in Clinical Improvement

One year after treatment, the clinical parameters, pocket depth, clinical attachment loss, bleeding on probing, and plaque score had improved significantly (Table 2).

**TABLE 2:** Periodontal treatment significantly reduces periodontitis.

	Before	After	*p*
Male/female	3/10		
Age (years)	46 ± 10	47 ± 10	
Pocket depth (mm)	3.96 ± 0.59	3.40 ± 0.40	< 0.001
Clinical attachment loss (mm)	3.99 ± 0.65	2.62 ± 0.39	< 0.001
Bleeding on probing (%)	64 ± 16	14 ± 7	< 0.001
Plaque (%)	53 ± 34	14 ± 12	< 0.001

*Note:* Mean ± standard deviation is shown, *n* = 13.

### 3.2 Sera Before and After Treatment Results in Similar Numbers of Osteoclasts

**FIGURE 1:**
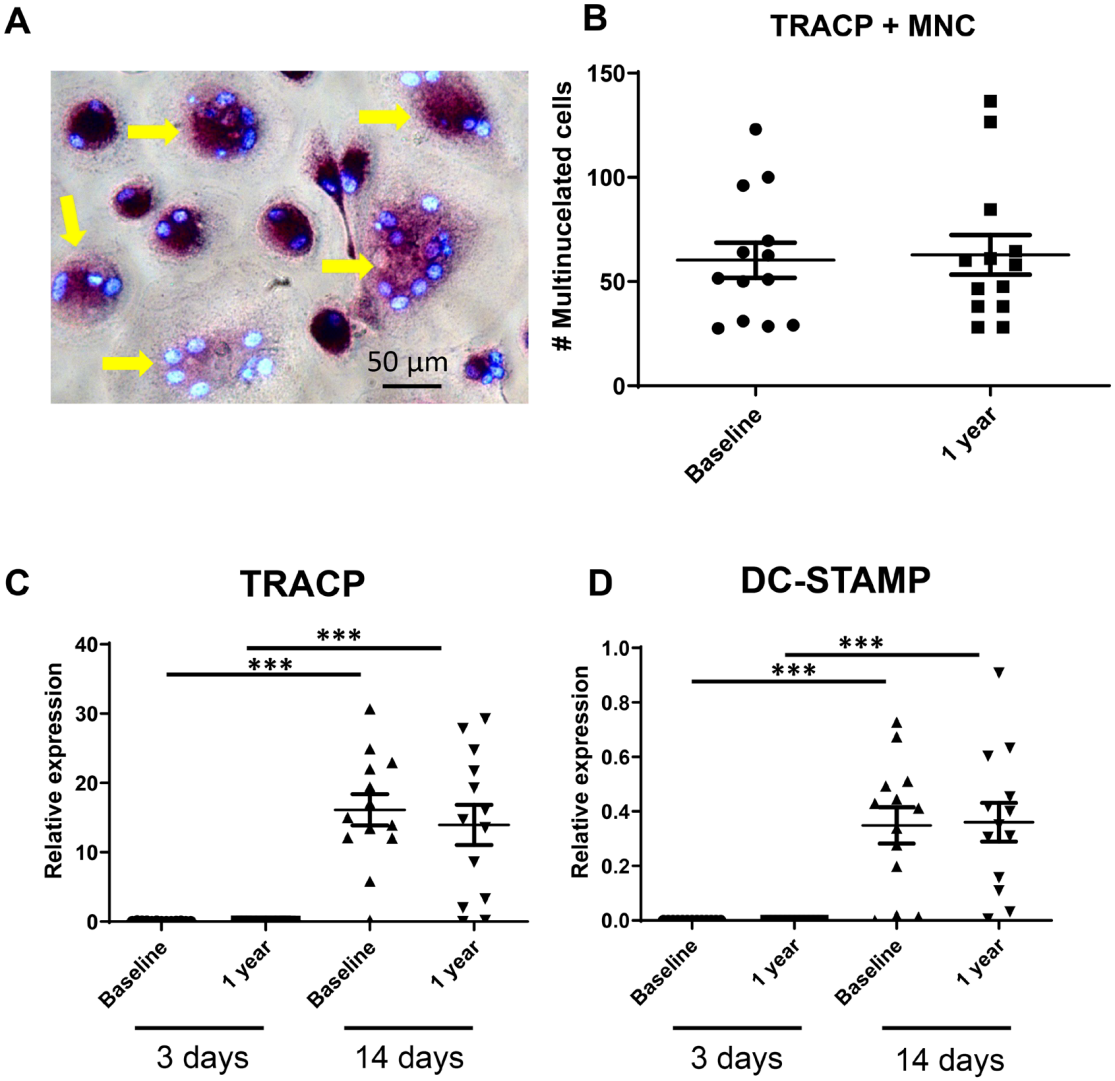
Priming of PDLF with serum at baseline and 1 year after successful periodontal treatment results in similar osteoclast numbers. PDLF were primed for 3 days with patient sera at baseline and 1 year after successful treatment. (A) Micrograph of TRACP‐positive multinucleated cells (osteoclasts), yellow arrows. (B) Osteoclast counts. TRACP‐positive multinucleated cells (> 2 nuclei per cell) were counted, no differences exists between at intake and 1 year later (*n* = 13) Red lines: Average. qPCR analysis of osteoclast markers (C) TRACP and (D) DC‐STAMP revealed no expression after the 3 day priming period and expression at day 14 after PBMCs were added as source of osteoclast precursors. ***p < 0.001, *t*‐test per serum condition, comparison between 3 days and 14 days. No differences existed between cell cultures at baseline and 1 year later.

Periodontal ligament fibroblasts were preincubated for 3 days with sera at baseline or 1 year later. PBMC were added at day three. Twenty‐one days later, the cells were fixed to assess the number of tartrate resistant acid phosphatase (TRAcP)‐positive multinucleated cells that formed. The number of TRAcP‐positive multinucleated cells (Figure 1A) was similar between serum at intake and 1 year later (Figure 1B). Out of the 13 paired samples, 6 donors displayed higher numbers of multinucleated cells after treatment with the baseline serum and 7 were higher after stimulation with the after 1 year serum. This was confirmed at the RNA level: osteoclast markers TRAcP (Figure 1C) and dendritic cell‐specific transmembrane protein DC‐STAMP (Figure 1D) were not detectable at day 3 and were at similar levels detected in osteoclast cultures that were treated with sera from baseline and 1 year after treatment. Likewise, no differences were observed between the baseline serum stimulation and the 1 year after treatment serum stimulation at the 3 days timepoint nor at 14 days for IL‐1β, IL‐6, IL‐17, M‐CSF, RANK, RANKL, OPG and TNF‐α (results not shown).

### 3.3 Unique Individual Serum Levels for IL‐6, MCP‐1 and TNF‐α at Intake and One Year Later

To explain the above findings, the composition of the sera was further investigated. From the pro‐osteoclastogenic cytokines (IL‐1β, IL‐6, IL‐17, MCP‐1 and TNF‐α) and anti‐osteoclastogenic cytokines (IFNγ, IL‐4, IL‐8 and IL‐10), only IL‐6, MCP‐1 and TNF‐α were detectable in the majority of the samples. For Luminex data for all measurable cytokines: see S1.

**FIGURE 2:**
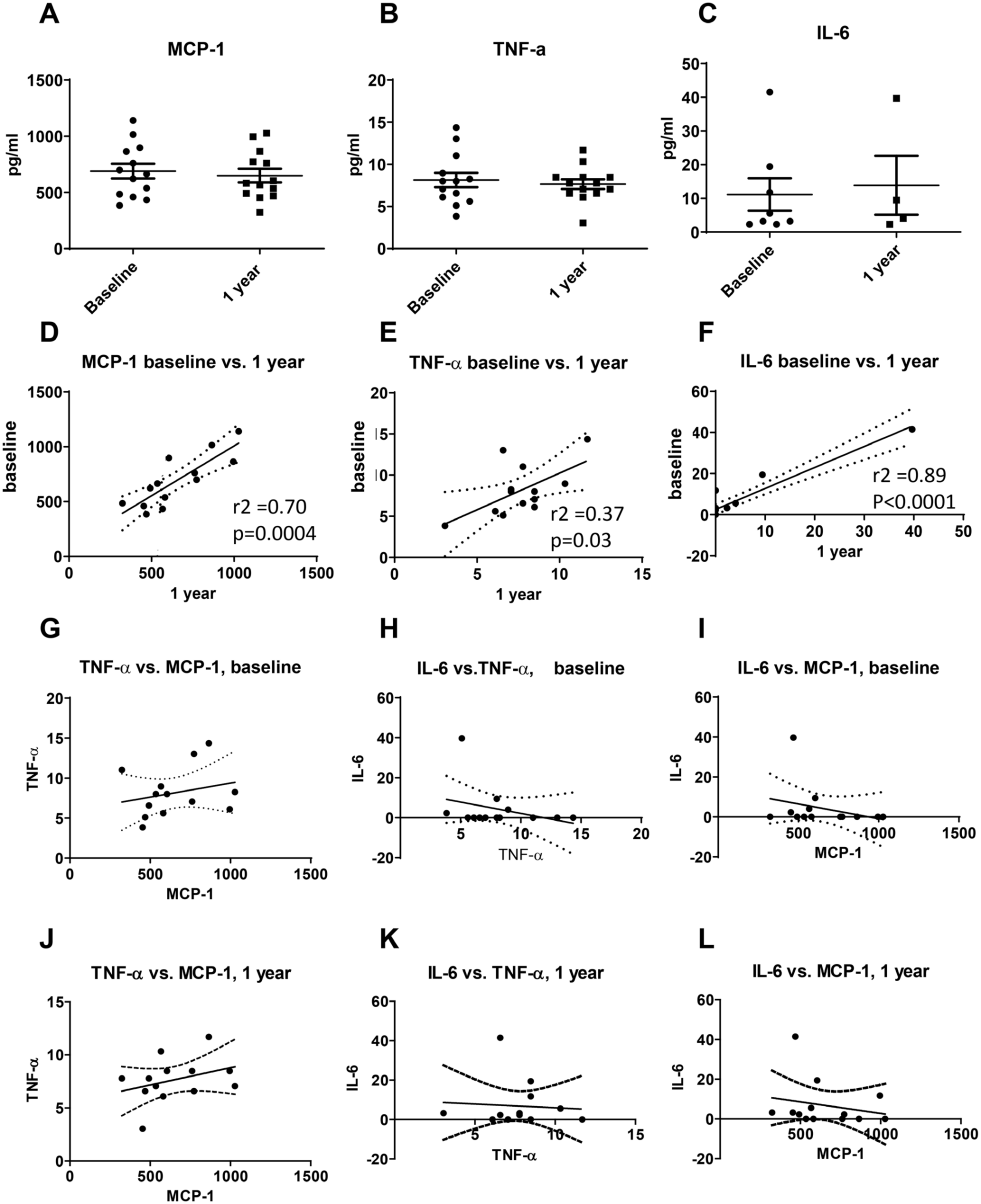
Serum measurements and correlations of MCP‐1, TNF‐α and IL‐6 at baseline and 1 year after successful periodontal treatment. No differences were observed between intake and 1 year later for MCP‐1 (A), TNF‐α (B) and IL‐6 (C). Serum measurement of MCP‐1 (D), TNF‐α (E) and IL‐6 (F) were similar at intake and 1 year later after successful treatment and correlated strongly per individual at baseline versus after 1 year of successful treatment. At intake (G‐I) or 1 year after (J–L), nor TNF‐α and MCP‐1 (G, J), nor IL‐6 and TNF‐α (H, K) nor IL‐6 and MCP‐1 levels (I, L) correlated. (*n* = 13).

Further analysis revealed that these serum levels at intake and after one year were comparable for the 13 samples for MCP‐1 (Intake: 689 ± 239 (S.D.) pg/mL; 1 year: 650 ± 60 (S.D.) pg/mL) (Figure 2A), TNF‐α (Intake: 8.2 ± 0.9 (S.D.) pg/mL; 1 year: 7.7 ± 0.6 (S.D.) pg/mL) (Figure 2B) and IL‐6 (Intake: 11.1 ± 4.8 (S.D.) pg/mL; 1 year: 13.9 ± 8.7 (S.D.) pg/mL) (Figure 2C). A further analysis revealed that concentrations at intake versus at one year after treatment of MCP‐1 (Figure 2D), TNF‐α (Figure 2E) and IL‐6 (Figure 2F) significantly correlated per patient (*p*‐values between 0.0001 and 0.03; r2 values between 0.37 and 0.89). A further analysis of these osteoclastogenic cytokines showed that these three markers did not correlate with each other, either at intake (Figure 2G–I) or 1 year after treatment (Figure 2J–L). This implies that the concentration of these cytokines is independent of each other: each individual has her or his own unique serum composition of cytokines that is constant, at least as assessed in this 1 year period.

## 4 Discussion

**FIGURE 3:**
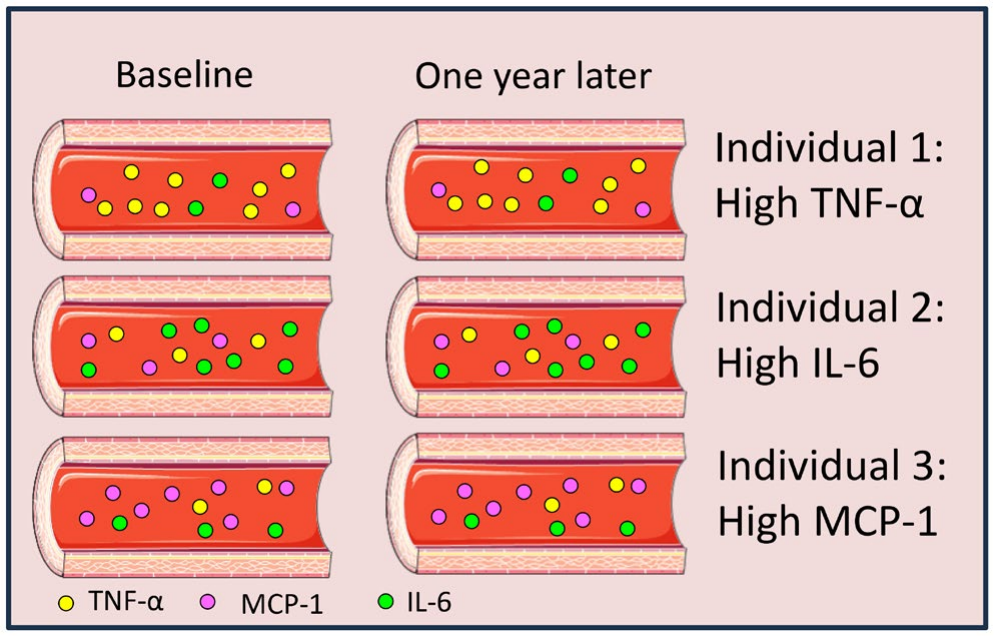
Graphic presentation of the concepts of cytokine equilibrium and cytokine signature. Individuals before and 1 year after treatment present with similar levels of MCP‐1, TNF‐α, and IL‐6. This is cytokine equilibrium. MCP‐1, TNF‐α, and IL‐6 are not correlated per individual, meaning that some individuals may present with relatively high levels of one of the cytokines with lower levels of the other cytokines. This is cytokine signature. Together, this finetuning of cytokine levels makes the individual suited for the serum needs of the own immune system. Graphics of blood vessels are from biomedical cartoons library that is freely available through Servier (smart.servier.com).

The present study initially addressed whether priming of periodontal ligament fibroblasts with serum from periodontitis patients at intake and 1 year after successful treatment would result in fewer osteoclasts. This was inspired by a similar set‐up for rheumatoid arthritis patients, where more osteoclasts were formed when osteoblasts were primed with serum from active RA compared to serum from patients in remission [8]. We therefore primed the periodontal equivalent, PDLFs, with periodontitis patients' sera, choosing the best responding 13. Also, in acute inflammatory diseases, a steep rise in inflammatory cytokines is usually seen. The rationale of the current hypothesis is that periodontitis, an inflammatory disease which causes destruction of alveolar bone, could show higher plasma levels of specific inflammatory markers in comparison with patients without periodontitis [16]. Our study, however, displayed no differences in osteoclast formation, in parallel with similar levels of osteoclast genes TRAcP and DC‐STAMP. In search of an explanation for these results, the cytokine serum composition was determined and showed that the majority of the 9 cytokines, except for MCP‐1, TNF‐α, and IL‐6, were either detectable in a few samples or not detectable or expressed. Moreover, when plotting the values per patient at baseline and after treatment, the values of MCP‐1, TNF‐α, and IL‐6 correlated strongly. This implies that, over the timespan of 1 year, individuals have a relatively constant serum level of these inflammatory cytokines that is unique to them, regardless of periodontal treatment. We can thus speculate that everyone might have an individual serum cytokine signature (Figure 3).

In order to try to generalise the concept of a cytokine equilibrium (i.e.,: similar levels at baseline and 1 year later), one could wonder whether the found levels of cytokines are typical for (treated) periodontitis patients. Average levels of MCP‐1 in the present study were approximately 700 pg/mL, which is considerably higher than the control groups [17] (209 pg/mL) and [18] (263 pg/mL) in previous studies. MCP‐1 levels could therefore reflect the periodontitis state and the chronic disease after treatment, which, in agreement with the present study, remains high after treatment. In contrast to the elevated MCP‐1 levels, however, average levels of TNF‐α in the present study were around 8 pg/mL, comparable with 6.0 in a large control cohort of 126 [19] and comparable to Lee et al.'s [20], where 5 pg/mL is reported for healthy controls (*n* = 29). The IL‐6 levels in our study were 10 pg/mL. A meta‐analysis of healthy control values noted 5.12 pg/mL, albeit that large variations were noticed [21]. Anti‐inflammatory cytokines IL‐10 and IL‐4 were not detectable in our Luminex assays. Though not the scope of this article, some of the leukocytes in blood may have an altered activity, for instance neutrophils. A recent systematic review found that patients with periodontitis showed a peripheral pro‐inflammatory neutrophil profile with elevated production of reactive oxygen species (ROS), in comparison with controls [22]. However, studies exploring a systemic cytokine profile did not show consistent results. For instance, Branco‐de Almeida et al. compared 17 cytokines present in gingival crevicular fluid and in plasma of local aggressive periodontitis, related healthy siblings and unrelated healthy controls [23]. For gingival crevicular fluid, 15 out of 17 markers differed between the periodontitis group and the two types of controls. Plasma levels however, differed for only 8 out of 17 cytokines. Relevant for the present study is that especially MCP‐1 and IL‐6 did not differ between periodontitis and controls. Whether or not periodontal pathology is reflected systemically is an ongoing debate. A recent meta‐analysis revealed that, when looking at a time‐window of on average 3 months, that serum levels of IL‐1β, TNF‐α and IL‐6 lower after non‐surgical periodontal treatment [24]. Zhang et al., studying mainly obesity and periodontitis, found lower serum levels for IL‐6 at 3 months [25]. Another study compared leukocyte cell composition and cytokines present in plasma for a group of chronic and aggressive periodontitis and healthy controls. No differences in blood composition regarding all major cell types were detected and no differences in cytokines TNF‐α, IL‐4, IL‐6, IL‐10, IL‐33, IL‐17F [26] were observed. Likewise, in a comparison of plasma levels between localised aggressive periodontitis, generalised aggressive periodontitis and healthy controls, no differences were found between the groups for cytokines IL‐1α, IL‐1β, IL‐1RA, IL‐6 and IL‐10 [27]. Others, however, found significant differences of many markers between serum from periodontitis patients and controls, among them MCP‐1 and IL‐6 [28]. The recent systematic review by Amad et al. confirm that MCP‐1 and TNF‐α levels are higher in sera from periodontitis patients than in healthy controls, differences of IL‐6 levels differed among studies [29]. These studies, however, focused on periodontitis versus health. The present study describes cytokines levels before and after treatment. To summarise the above studies: cytokine levels in gingival crevicular fluid seems a better indicator of periodontitis than some serum values [23]. The above studies found only limited [23] or no differences [24, 25] between cytokines present in periodontitis patients' blood and that of healthy controls. These studies, that show no differences between periodontitis patients and healthy controls in terms of cytokine serum levels, then feed the idea that the cytokine signature (the individual levels of the various cytokines) and the cytokine equilibrium (the stable concentration of certain cytokines over a one‐year period), could account for healthy individuals as well.

Two studies from the same group compared leukocyte composition [30] and cytokine levels [31] before and after treatment. Medara et al. monitored leukocyte composition of good, moderate and poor responders to therapy at baseline and at 3, 6 and 12 months [30]. No differences were observed between baseline up to 12 months for any of the groups for nearly all different T‐cell populations. The only difference was found in a higher prevalence of Fox‐p3 T‐cells (Tregs), where significantly higher percentages were seen in good and moderate responders after treatment. In the paper that compared cytokine levels for 54 periodontitis patients and 40 healthy controls, cytokines IL‐1β, IL‐4, IL‐6, IL‐10, IL‐17A, IL‐17F, IL‐21, IL‐22, IL‐23, IL‐25, IL‐31, IL‐33, IFN‐γ, sCD40L and TNF‐α were measured in saliva and serum at baseline, 3, 6 and 12 months after treatment. Where saliva concentrations of these cytokines were a good indicator of response, fewer differences were seen in the serum [31]. When summarising the above studies in the context of serum measurements before and after treatment, crevicular fluid and saliva parameters better reflect the response to treatment than serum cytokine measurements. Given the heterogeneity between donors of responses of PBMCs in the blood to cytokines, a next level of interpreting the results would also consider whether PBMCs from patients would differentially respond to IL‐6, MCP‐1 and TNF‐α.

The original hypothesis, that PDLFs would generate more osteoclasts when primed with serum from periodontitis patients before treatment had to be rejected. Analysis of the sera before and after treatment unveiled a possible explanation for this, since the measurable cytokines did not reveal differences before and after treatment. On the one hand levels of the three cytokines TNF‐α, IL‐6 and MCP‐1 correlate with before treatment and 1 year after, but on the other hand the three cytokines do not correlate with the other two cytokines before or after treatment, it is tempting to speculate more on the cytokine signature and equilibrium. Cytokine signature, the individual cytokine composition in peripheral blood, therefore, seemed to be shaped per individual at concentrations per cytokine that are constant over 1 year, independent of treatment, that is tailored to and probably fit for the specific individual. The consequences of cytokine signature (see Figure 3) could be that individuals may have higher levels of TNF‐α, or IL‐6 or MCP‐1, together with a variation of other cytokines. Though the differences in cytokine composition between individuals are often subtle, and though there are overlapping functions between cytokines, it is tempting to speculate about how the existing cytokine signature will cause specialization of immune responses between individuals. Connecting biological function of the cytokines, individuals with higher TNF‐α levels could be slightly skewed toward activation of macrophages, fever induction and killing of pathogens [30]. Individuals with higher levels of IL‐6 could then be more prepared for T‐cell activation, fever induction, haematopoietic stem cell maturation and tissue destruction [32]. Finally, individuals with higher levels of MCP‐1 have an immune system that is more equipped on recruitment of immune cells [33].

## 5 Conclusions

Cytokine equilibrium, the relatively similar levels of cytokines over a longer period, together with cytokine signature, the individual periodontitis patients adapts the levels of cytokines, contribute to the best fit of the immune system over a longer period, here 1 year, for the individual periodontitis patient. Whether this intriguing phenomenon exists also for healthy individuals and whether it exists over a longer period and for more cytokines should be the aim of a next study. A larger cohort is needed for more definite answers on cytokine signature and equilibrium.

## Funding

The authors have nothing to report.

## Conflicts of Interest

The authors declare no conflicts of interest.

## Supporting information


**Appendix S1:** sji70094‐sup‐0001‐AppendixS1.docx.

## Data Availability

The data that support the findings of this study are available from the corresponding author upon reasonable request.

## References

[1] P. P. Souza and U. H. Lerner , “The Role of Cytokines in Inflammatory Bone Loss,” Immunological Investigations 42, no. 7 (2013): 555–622, 10.3109/08820139.2013.822766. 24004059

[2] A. Cekici , A. Kantarci , H. Hasturk , and T. E. Van Dyke , “Inflammatory and Immune Pathways in the Pathogenesis of Periodontal Disease,” Periodontology 2000 64, no. 1 (2014): 57–80, 10.1111/prd.12002. 24320956 PMC4500791

[3] M. Zhang , Y. Liu , H. Afzali , and D. T. Graves , “An Update on Periodontal Inflammation and Bone Loss,” Frontiers in Immunology 15 (2024): 1385436, 10.3389/fimmu.2024.1385436. 38919613 PMC11196616

[4] D. Sokos , V. Everts , and T. J. de Vries , “Role of Periodontal Ligament Fibroblasts in Osteoclastogenesis: A Review,” Journal of Periodontal Research 50, no. 2 (2015): 152–159, 10.1111/jre.12197. 24862732

[5] T. J. de Vries , I. El Bakkali , T. Kamradt , G. Schett , I. D. C. Jansen , and P. D'Amelio , “What Are the Peripheral Blood Determinants for Increased Osteoclast Formation in the Various Inflammatory Diseases Associated With Bone Loss?,” Frontiers in Immunology 10 (2019): 505, 10.3389/fimmu.2019.00505. 30941138 PMC6434996

[6] G. Schett , “Review: Immune Cells and Mediators of Inflammatory Arthritis,” Autoimmunity 41, no. 3 (2008): 224–229, 10.1080/08916930701694717. 18365836

[7] F. Zamri and T. J. de Vries , “Use of TNF Inhibitors in Rheumatoid Arthritis and Implications for the Periodontal Status: For the Benefit of Both?,” Frontiers in Immunology 11 (2020): 591365, 10.3389/fimmu.2020.591365. 33193432 PMC7646519

[8] J. L. Pathak , N. Bravenboer , P. Verschueren , et al., “Inflammatory Factors in the Circulation of Patients With Active Rheumatoid Arthritis Stimulate Osteoclastogenesis via Endogenous Cytokine Production by Osteoblasts,” Osteoporosis International 25, no. 10 (2014): 2453–2463, 10.1007/s00198-014-2779-1. 25027107

[9] M. Capri , S. Fronterrè , S. Collura , et al., “Circulating CXCL9, Monocyte Percentage, Albumin, and C‐Reactive Protein as a Potential, Non‐Invasive, Molecular Signature of Carotid Artery Disease in 65+ Patients With Multimorbidity: A Pilot Study in Age,” Frontier Endocrinology (Lausanne) 15 (2024): 1407396, 10.3389/fendo.2024.1407396. PMC11300199 39109084

[10] I. Savulescu‐Fiedler , R. Mihalcea , S. Dragosloveanu , et al., “The Interplay Between Obesity and Inflammation,” Life 14, no. 7 (2024): 856, 10.3390/life14070856. 39063610 PMC11277997

[11] N. G. F. M. Beukers , N. Su , G. J. M. G. van der Heijden , and B. G. Loos , “Periodontitis Is Associated With Multimorbidity in a Large Dental School Population,” Journal of Clinical Periodontology 50, no. 12 (2023): 1621–1632, 10.1111/jcpe.13870. 37658672

[12] S. Bizzarro , U. van der Velden , W. J. Teeuw , V. E. A. Gerdes , and B. G. Loos , “Effect of Periodontal Therapy With Systemic Antimicrobials on Parameters of Metabolic Syndrome: A Randomized Clinical Trial,” Journal of Clinical Periodontology 44, no. 8 (2017): 833–841, 10.1111/jcpe.12763. 28621003 PMC5599971

[13] T. J. de Vries , T. Schoenmaker , N. Wattanaroonwong , et al., “Gingival Fibroblasts Are Better at Inhibiting Osteoclast Formation Than Periodontal Ligament Fibroblasts,” Journal of Cellular Biochemistry 98, no. 2 (2006): 370–382, 10.1002/jcb.20795. 16440316

[14] T. J. de Vries , T. Schoenmaker , D. Aerts , et al., “M‐CSF Priming of Osteoclast Precursors Can Cause Osteoclastogenesis‐Insen Sitivity, Which Can Be Prevented and Overcome on Bone,” Journal of Cellular Physiology 230, no. 1 (2015): 210–225, 10.1002/jcp.24702. 24962140

[15] E. Steemers , W. M. I. Talbi , J. M. A. Hogervorst , T. Schoenmaker , and T. J. de Vries , “IL‐1 Receptor Antagonist Anakinra Inhibits the Effect of IL‐1β‐ Mediated Osteoclast Formation by Periodontal Ligament Fibroblasts,” Biology 14, no. 3 (2025): 250, 10.3390/biology14030250. 40136507 PMC11939651

[16] S. Paraskevas , J. D. Huizinga , and B. G. Loos , “A Systematic Review and Meta‐Analyses on C‐Reactive Protein in Relation to Periodontitis,” Journal of Clinical Periodontology 35, no. 4 (2008): 277–290, 10.1111/j.1600-051X.2007.01173.x. 18294231

[17] W. Gao , Y. Xu , J. Liang , et al., “Serum CC Chemokines as Potential Biomarkers for the Diagnosis of Major Depressive Disorder,” Psychology Research and Behavior Management 15 (2022): 2971–2978, 10.2147/PRBM.S384267. 36310625 PMC9604417

[18] M. M. Hayatbakhsh , A. Gowhari Shabgah , S. Pishgouyi , J. Tavakol Afshari , H. Zeidabadi , and M. Mohammadi , “The Serum Levels of CCL2 and CCL16 Expression in Patients With Irritable Bowel Syndrome,” Reports of Biochemistry and Molecular Biology 8, no. 1 (2019): 9–14. 31334281 PMC6590941

[19] G. Li , W. Wu , X. Zhang , et al., “Serum Levels of Tumor Necrosis Factor Alpha in Patients With IgA Nephropathy Are Closely Associated With Disease Severity,” BMC Nephrology 19, no. 1 (2018): 326, 10.1186/s12882-018-1069-0. 30428849 PMC6236996

[20] S. J. Lee , Z. Li , B. Sherman , and C. S. Foster , “Serum Levels of Tumor Necrosis Factor‐Alpha and Interleukin‐6 in Ocular Cicatricial Pemphigoid,” Investigative Ophthalmology & Visual Science 34, no. 13 (1993): 3522–3525. 8258509

[21] E. A. Said , I. Al‐Reesi , N. Al‐Shizawi , et al., “Defining IL‐6 Levels in Healthy Individuals: A Meta‐Analysis,” Journal of Medical Virology 93, no. 6 (2021): 3915–3924, 10.1002/jmv.26654. 33155686

[22] R. A. Irwandi , S. O. Kuswandani , S. Harden , D. Marletta , and F. D'Aiuto , “Circulating Inflammatory Cell Profiling and Periodontitis: A Systematic Review and Meta‐Analysis,” Journal of Leukocyte Biology 111, no. 5 (2022): 1069–1096, 10.1002/JLB.5RU1021-524R. 35199874

[23] L. S. Branco‐de‐Almeida , Y. Cruz‐Almeida , Y. Gonzalez‐Marrero , et al., “Local and Plasma Biomarker Profiles in Localized Aggressive Periodontitis,” JDR Clinical & Translational Research 2, no. 3 (2017): 258–268, 10.1177/2380084417701898. 28879248 PMC5576058

[24] M. Cardisciani , S. Di Nicolantonio , S. Altamura , E. Ortu , R. Del Pinto , and D. Pietropaoli , “Temporal Dynamics of Early Inflammatory Markers After Professional Dental Cleaning: A Meta‐Analysis and Spline‐Based Meta‐Regression of TNF‐α, IL‐1β, IL‐6, and (Hs)CRP,” Frontiers in Immunology 16 (2025): 1634622, 10.3389/fimmu.2025.1634622. 40948802 PMC12423065

[25] Y. Zhang , R. Jia , Y. Zhang , et al., “Effect of Non‐Surgical Periodontal Treatment on Cytokines/Adipocytokines Levels Among Periodontitis Patients With or Without Obesity: A Systematic Review and Meta‐Analysis,” BMC Oral Health 23 (2023): 717. 37798684 10.1186/s12903-023-03383-3 PMC10552206

[26] W. C. Cheng , F. Saleh , B. Abuaisha Karim , F. J. Hughes , and L. S. Taams , “Comparative Analysis of Immune Cell Subsets in Peripheral Blood From Patients With Periodontal Disease and Healthy Controls,” Clinical and Experimental Immunology 194, no. 3 (2018): 380–390, 10.1111/cei.13205. 30120837 PMC6231023

[27] A. Havemose‐Poulsen , L. K. Sørensen , K. Stoltze , K. Bendtzen , and P. Holmstrup , “Cytokine Profiles in Peripheral Blood and Whole Blood Cell Cultures Associated With Aggressive Periodontitis, Juvenile Idiopathic Arthritis, and Rheumatoid Arthritis,” Journal of Periodontology 76, no. 12 (2005): 2276–2285, 10.1902/jop.2005.76.12.2276. 16332240

[28] M. Wänman , S. Betnér , A. Esberg , et al., “The PerioGene North Study Uncovers Serum Proteins Related to Periodontitis,” Journal of Dental Research 103, no. 10 (2024): 999–1007, 10.1177/00220345241263320. 39101637 PMC11402264

[29] P. Ahmad , J. Slots , and W. L. Siqueira , “Serum Cytokines in Periodontal Diseases,” Periodontology 2000 (2025), 10.1111/prd.12629. PMC12842884 40995683

[30] N. Medara , J. C. Lenzo , K. A. Walsh , N. M. O'Brien‐Simpson , E. C. Reynolds , and I. B. Darby , “Peripheral T Helper Cell Profiles During Management of Periodontitis,” Journal of Clinical Periodontology 48, no. 1 (2021): 76–90, 10.1111/jcpe.13389. 33051896

[31] N. Medara , J. C. Lenzo , K. A. Walsh , I. B. Darby , N. M. O'Brien‐Simpson , and E. C. Reynolds , “T Helper 17 Cell‐Related Cytokines in Serum and Saliva During Management of Periodontitis,” Cytokine 134 (2020): 155186, 10.1016/j.cyto.2020.155186. 32717609

[32] C. A. Hunter and S. A. Jones , “IL‐6 as a Keystone Cytokine in Health and Disease,” Nature Immunology 16, no. 5 (2015): 448–457, 10.1038/ni.3153. 25898198

[33] S. L. Deshmane , S. Kremlev , S. Amini , and B. E. Sawaya , “Monocyte Chemoattractant Protein‐1 (MCP‐1): An Overview,” Journal of Interferon & Cytokine Research 29, no. 6 (2009): 313–326, 10.1089/jir.2008.0027. 19441883 PMC2755091

