# Supplementary material for: Similar Individual Serum Levels of MCP‐1, TNF‐α, and IL‐6 From Periodontitis Patients Before and 1 Year After Treatment
Source: Scand J Immunol. 2026 Jan 24;103(2):e70094. doi: 10.1111/sji.70094 (PMC12831644; doi:10.1111/sji.70094)
Supplement: Supplementary file 1 — Appendix S1: sji70094‐sup‐0001‐AppendixS1.docx. [file SJI-103-e70094-s001.docx]

**Supplementary file.**

Luminex data.

Table below shows the raw data of all Luminex measurements of all cytokines that were measured. 0 means below the detection range of the standard curve. * means extrapolated from the standard curve. Such data were considered unreliable, and cytokine data set containing extrapolated data was excluded from further analysis.
